# A retrospective single‐institute study reveals a vertical gradient of the density of cutaneous melanoma from head to toe

**DOI:** 10.1002/ski2.463

**Published:** 2024-11-02

**Authors:** Meryl Musicante, James Ferrer, Jianjian Lin, Tejesh Surendr Patel, Feng Liu‐Smith

**Affiliations:** ^1^ College of Medicine University of Tennessee Health Science Center Memphis Tennessee USA; ^2^ Department of Dermatology College of Medicine University of Tennessee Health Science Center Memphis Tennessee USA; ^3^ Department of Preventive Medicine College of Medicine University of Tennessee Health Science Center Memphis Tennessee USA

## Abstract

**Background:**

The bodily distribution of melanoma is frequently reported without consideration of the skin surface area, which could be misleading in melanoma risk regarding anatomical sites.

**Objectives:**

To gain insights into the melanoma distribution on the body surface when the body surface area is considered.

**Methods:**

Cutaneous melanoma data were extracted from a single dermatopathology laboratory, and the relative density from each body site was calculated by taking into consideration the skin surface area. Data from a previous publication were analyzed as a validation. Surveillance, Epidemiology and End Results Program data were also used for comparison.

**Results:**

Relative tumour density (RTD) of melanoma in men and women exhibits a moderate head‐to‐toe linear gradient, with the upper body sites showing higher density than the lower body sites in general. In particular, the ear and face show the highest RTD while the least UVR (ultraviolet radiation)‐exposed buttock, abdomen and groin have the lowest, followed by the thigh and lower legs. The trend is similar in both sexes, but more obvious for men.

**Conclusions:**

It was well documented that the trunk and lower legs are the most frequently diagnosed sites for men and women, respectively. However, when the surface area is considered, the melanoma distribution exhibits a rough head‐to‐toe gradient, which perhaps reflects a combined effect of solar UVR and clothing coverage. UVR protection on the face and ear should be emphasized as these are the sites with the highest RTDs.

## Graphical abstract

Using melanoma density as an indicator, we show that there is a moderate head‐to‐toe distribution of cutaneous melanoma, which may reflect a combined effect of sun exposure and protection from hair and clothing. These results emphasize protection on the ear and face of both men and women as these sites show the highest density of tumours.

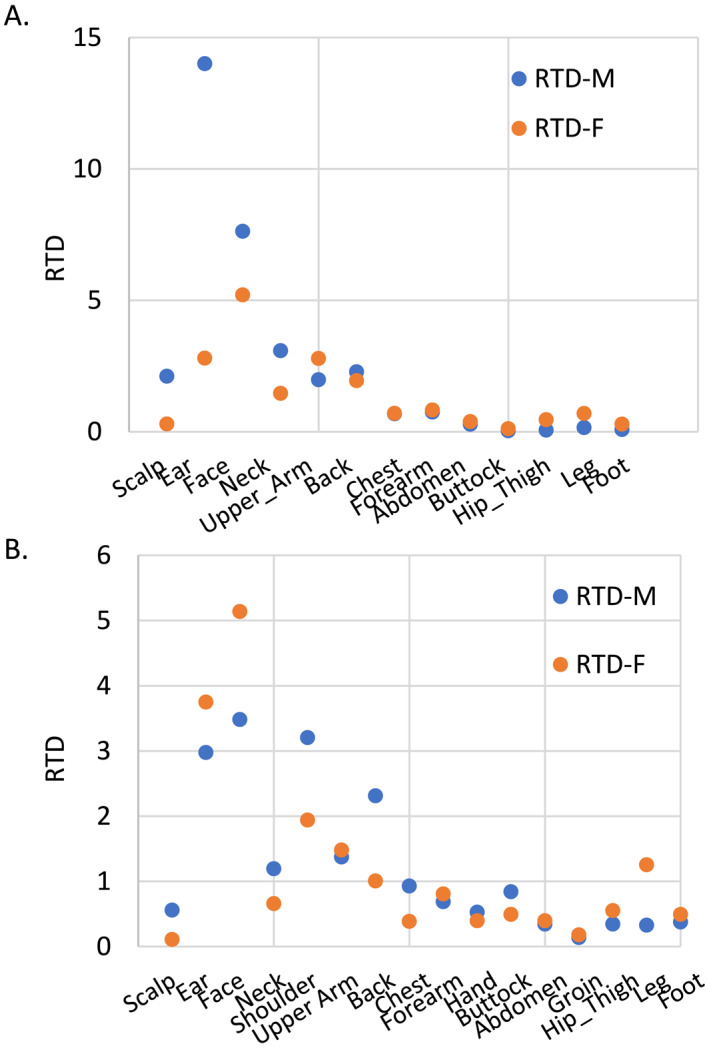

**What is already known?**

- Cutaneous melanoma distribution is uneven across different body sites.
- Women's melanomas are mainly found in their legs while men's melanoma are found in their trunk.

**What does this study add?**

- By calculating the density of melanoma tumours using relative body surface area, the distribution in the legs or trunk is no longer considered the most prominent; instead, the ear and face show the highest density of tumours.
- These results suggest that we must put more emphasis on the protection of our ear and face. Overall, melanoma distribution follows a head‐to‐toe gradient.

## 1 INTRODUCTION

Cutaneous malignant melanoma (CMM) is the deadliest form of skin cancer with increasing incidence worldwide.^1^, ^2^, ^3^, ^4^ In 2024, it is estimated that 59 170 men and 41 470 women will be diagnosed with melanoma in the United States, with 5430 and 2860 deaths, respectively.^5^ The cause for such increasing trends remains largely unknown, with uncertainty of host‐specific factors in addition to the role of ultraviolet radiation (UVR) in the pathogenesis of melanoma.^6^, ^7^, ^8^

Worldwide, men of all skin types show higher incidence rates of melanoma than women.^9^ However, there is a significant age‐dependent sex difference. Younger women and older men are at higher risk than their peers.^10^ Anatomic locations of the tumours are also different between sexes.^11^, ^12^, ^13^ The most common location for melanoma diagnosis on men and women is the trunk and the legs, respectively, a finding that is characteristic of the intermittent exposure theory.^14^, ^15^ However, when body surface areas are taken into account, areas of chronic exposure, such as the face, have the highest density of melanoma.^15^ This pattern is most consistent with the chronic sun exposure theory of melanoma pathogenesis.

Individual‐level data from most cancer registries is not specific on anatomic sites and can be missing key information. The US Surveillance, Epidemiology and End Results Program (SEER) database limits the body to several anatomic sites: the scalp, neck, face, ear, trunk, shoulder and upper extremities and hip and lower extremities. The CI5plus data (Cancer Incidence in Five Continents, Volume XI) from the International Agency for Research on Cancer only provides aggregated data. There are some previous reports on the anatomic distribution of melanoma, which provides very interesting insight into the relationship between CMM and UVR.^15^, ^16^

CMMs from different body locations exhibit dramatic sex difference,^17^, ^18^ have different etiological pathways^19^ and also show different survival patterns.^20^ Hence, study site‐specific distribution of melanoma may bring insightful understanding of CMM in these aspects. In this paper, we asked whether the melanoma exhibited a head to toe density through a detailed analysis of precise melanoma anatomic location data from one major academic institution, and we assessed the relationship between body site, age and sex, as well as their correlation with tumour characteristics such as the Breslow depth and status of ulceration.

## 2 MATERIALS AND METHODS

### 2.1 Data extraction

Following institutional review board approval (UTHSC IRB 20‐07559‐XP), the age, sex and body site for all melanoma diagnoses (including melanomas in situ) were retrospectively collected from the UTMG (University of Tennessee Medical Group) dermatopathology archived from 1 January 2013 to 14 October 2021. The initially collected data include a case number (de‐identified), date of diagnosis, age of diagnosis, sex and the medical notes containing details regarding diagnosis and surgery, margins, the Breslow thickness and ulceration status and whether the melanoma is in situ or not. Occasionally the note might indicate ‘no melanoma found’, in which case the data entry was removed.

When the raw data were extracted, a ‘Site’ variable was created to give detailed sites of tumours, such as the left cheek, right or middle abdomen or upper or lower chest. Any site entry containing the words ‘scalp’, ‘crown’, ‘occipital’ or ‘vertex’ is grouped into the ‘scalp’ location. Any site entry containing one of the following words is grouped into the ‘face’ location: cheek, jaw, jawline, eyelid, brow, eyebrow, lip, forehead, chin, nose, glabella, alar crease, alar groove, buccal cheek, temple, zygoma, facial mass, infraorbital rim, infraorbital skin, malar, mandibular, submandibular, medial canthus, nares, nasal (or nose), canthus, parietal, sideburn and face. Any site entry containing one of the following words is grouped into the ‘ear’ location: auricular, ear, earlobe, concha, helix, pinna rim, tragus, mastoid and scapha. Any site entry containing the word ‘abdomen’ is grouped into the ‘abdomen’ location plus one site that is located in the umbilicus. Any site entry containing the word ‘neck’ is grouped into the neck location. Any site entries containing one of the following words are grouped into ‘chest’: chest, breast, clavicle, clavicular, infraclavicular, sternum and xiphoid. Any site entry containing the word ‘back’ is grouped into the back location. Any site entry containing the following words is grouped into the ‘upper arm’ location: upper arm, shoulder, deltoid, antero‐lateral, triceps and biceps. Any site entry containing the following words is grouped into the ‘forearm/hand’ location: forearm, fossa, lower arm, distal arm, hand, palm and finger. Any site entry containing the following words is grouped into the buttock/groin location: buttock, anal, perirectal, pubic, groin, inguinal, labia and scrotum. Any site entry containing the following words is grouped into the thigh/hip location: thigh, knee and hip. Any site entry containing the following words is grouped into the lower leg location: lower leg, shin, calf, tibial, ankle and poplitaeal. Any site entry containing the following words is grouped into the foot location: foot, heel, toe and metatarsal.

A second dataset was obtained from Bulliard et al. published in 2007.^21^ Briefly, 1658 primary malignant melanoma cases (1995–2002) were collected in Switzerland for relative melanoma density calculation based on body site surface areas. Among the cases, there were 874 males and 1014 females. This dataset was used as the primary validation set.

The third dataset was SEER data, which was accessed using the SEERStat software (Version 8.4.2) with individual level of data. Only malignant melanoma (International Classification of Diseases for Oncology code 3) was included (melanoma in situ was excluded). Melanoma definition and data download follow a previous study.^22^ This dataset was used as a second validation set.

### 2.2 Calculation of RTD

To calculate relative tumour densities (RTDs), the sum of tumour numbers in each anatomic site was divided by total tumour numbers to give the percentage of case numbers in each sex in the UTMG dataset, which was then divided by the average percentage of body surface area (BSA) for each site of US men and women from previous studies.^21^, ^23^ Specifically, RTD = (% case numbers on an anatomic site)/(% BSA of that anatomic site).

### 2.3 Statistical analysis

All statistical analysis was carried out using the Stata17 software (StataCorp LLC) unless otherwise stated. Age‐standardized incidence rates from SEER data were calculated using the US 2000 standard population. Chi‐squared test was used to compare ulceration rates while the Wilcoxon rank‐sum test or Kruskal–Wallis rank‐sum test was used to compare the Breslow depth of tumours between sex or among sites and age groups. For all tests, the significance levels are set at *p* < 0.05 (two‐sided). The missing data in the primary dataset are minimum for the main variable location (0.2%) and hence the missing data were simply removed for statistical analysis.

## 3 RESULTS

### 3.1 Baseline characteristics of cutaneous melanoma cases

Melanoma data were extracted from the UTMG database at the University of Tennessee Health Science Center at Memphis. A total of 7236 primary cutaneous melanomas were included from 2013 to 2021: 2757 (38%) female and 4479 (62%) male patients (Table 1, Table S1). There were 2318 invasive melanomas and 4918 melanomas in situ. The mean ages of diagnosis for females and males were 62.3 and 69.2, respectively. Among patients aged <50, melanoma was more common in females (20% of all female tumours) as compared to males (6% of all tumours in males) in that age group (Table 1). Furthermore, in the 0–39 age group, females make up 76% of cases while male cases only account for 24% (Table S1). In contrast, in the 70+ age groups, nearly 72% of cases occur in men while only 28% of cases occur in women (Table S1).

**TABLE 1:** Baseline characteristics of the cases.

	Female (*n* = 2757, 38.1%)	Male (*n* = 4479, 61.9%)	All (*n* = 7236)
Age			
Mean (±SD)	62.3 ± 14.8	69.2 ± 11.6	66.6 ± 13.3
0–39	233 (9%)	74 (2%)	307 (4%)
40–49	314 (11%)	196 (4%)	510 (7%)
50–59	530 (19%)	563 (13%)	1093 (15%)
60–69	768 (28%)	1328 (30%)	2096 (29%)
70–79	592 (22%)	1502 (34%)	2094 (29%)
≥80	320 (12%)	816 (18%)	1136 (16%)
Total	2757 (100%)	4479 (100%)	7236 (100%)
Site, *n* (%)			
Scalp	30 (1%)	347 (8%)	377 (5%)
Face	345 (13%)	818 (18%)	1163 (16%)
Ear	38 (1%)	312 (7%)	350 (5%)
Neck	96 (%)	331 (7%)	427 (6%)
Chest	194 (7%)	305 (7%)	499 (7%)
Abdomen	64 (2%)	77 (2%)	152 (2%)
Back	535 (19%)	1023 (23%)	1558 (224%)
Upper arm	616 (22%)	709 (16%)	1325 (18%)
Forearm/hand	252 (9%)	365 (8%)	617 (9%)
Buttock/groin	21 (0.7%)	8 (0.2%)	29 (0.5%)
Thigh/hip	241 (9%)	51 (1%)	292 (4%)
Lower leg	266 (10%)	98 (2%)	364 (5%)
Foot	55 (2%)	26 (0.6%)	81 (1%)
Missing	4 (0.2%)	9 (0.2%)	13 (0.2%)
Total	2757 (100%)	4479 (100%)	7236 (100%)
Melanoma category			
Melanoma in situ	1868 (38%)	3050 (62%)	4918 (100%)
Invasive melanoma	889 (38%)	1429 (62%)	2318 (100%)
Total	2757 (100%)	4479 (100%)	7236 (100%)
For invasive melanoma only			
Ulceration, *n* (%)			
No	626 (70%)	958 (67%)	1584 (68%)
Yes	142 (16%)	273 (19%)	415 (18%)
Missing	121 (10%)	198 (14%)	319 (14%)
Total	889 (100%)	1429 (100%)	2318 (100%)
Breslow depth [median]	0.6 mm	0.65 mm	0.6 mm
≤1.0 mm	596 (67%)	904 (63%)	1500 (65%)
1.01–2.0 mm	120 (14%)	220 (15%)	340 (15%)
2.01–4.0 mm	56 (6%)	138 (10%)	194 (8%)
≥4.01 mm	40 (5%)	58 (4%)	98 (4%)
Missing	77 (9%)	109 (8%)	186 (8%)
Total	889 (100%)	1429 (100%)	2318 (100%)

### 3.2 Distribution of melanoma in various anatomic locations

Melanoma location was divided into 13 sites to provide site‐specific incidence (Table 1). Thirteen patients did not have location data. Melanomas on the hand (*n* = 14), groin (*n* = 8) and hips (*n* = 4) are grouped with the forearm, buttock and thigh, respectively, due to the small numbers. In males, the most frequent location of melanoma was the back with 23% of melanomas, followed by the face and upper arms with 18% and 16%, respectively. In females, the most frequent locations were the upper arms and back with 22% and 19%, respectively.

### 3.3 Ulceration

Of the 2318 cases of invasive melanoma, 1999 contained ulceration data. Overall, there is a significant sex difference in ulceration rate, with 66% of all ulcerated cases occurring in men (*p* = 0.048, *χ* ^2^ test, Table 2). Rates of ulceration are not significantly different among different anatomic sites for women (*p* = 0.35, *χ* ^2^ test) but they are significantly different in men (*p* = 0.006, *χ* ^2^ test, Table 2). The buttock/groin and foot seem to have the highest rate of ulcerated tumours, but the case numbers are small (*n* < 10). The scalp (33%) and ear (27%) show the next highest ulceration rate in men. Most sites do not show sex difference in ulceration rates except for the buttock/groin (*p* = 0.019). However, as there are only 14 and 6 cases in women and men, respectively, for this site, this result may not reflect a true difference.

**TABLE 2:** Site and age‐specific differences in ulceration status.

	Female				Male				*p* values^a^
	Non‐ulcer (*n*)	Ulcerated (*n*)	Total (*n*)	Ulcer rate	Non‐ulcer (*n*)	Ulcerated (*n*)	Total (*n*)	Ulcer rate	
Site									
Scalp	7	0	7	0%	68	34	102	33%	0.066
Face	38	8	46	17%	86	24	110	22%	0.532
Ear	3	3	6	50%	52	19	71	27%	0.226
Neck	17	5	22	23%	49	11	60	18%	0.656
Chest	34	8	42	19%	53	16	69	23%	0.607
Abdomen	19	2	21	10%	27	5	32	16%	0.521
Back	136	32	168	19%	292	61	353	17%	0.622
Upper arm	140	37	177	21%	187	52	239	22%	0.834
Forearm/hand	51	11	62	18%	100	30	130	23%	0.399
Buttock/groin	12	2	14	14%	2	4	6	67%	0.019^b^
Thigh/hip	73	9	82	11%	20	5	25	20%	0.241
Lower leg	82	17	99	17%	34	8	42	19%	0.790
Foot	16	7	23	30%	4	5	9	56%	0.187
Total	628	141	769	18%	974	274	1248	22%	**0.048**
*p* values^c^	0.35				**0.006**				
Age									
0–39	57	19	76	25%	22	10	32	31%	0.503
40–49	89	15	104	14%	55	18	73	25%	0.097
50–59	125	20	145	14%	144	37	181	20%	0.135
60–69	175	30	205	15%	287	76	363	21%	0.066
70–79	114	31	145	22%	308	90	398	23%	0.697
≥80	71	27	98	28%	158	43	201	21%	0.287
Total	631	142	773	18%	974	274	1248	22%	**0.048**
*p* values^c^	**0.018**				0.77				

*Note*: Bold values indicate the significant p values, with two‐sided alpha 〈0.05.

^a^ *p* values for sex difference in ulceration rates in different sites (top) or age groups (bottom).

^b^ *p* value is not significant if Bonferroni correction is applied (*α* = 0.0038).

^c^ *p* values for site (top) or age (bottom) difference in ulceration rate in each sex.

**FIGURE 1:**
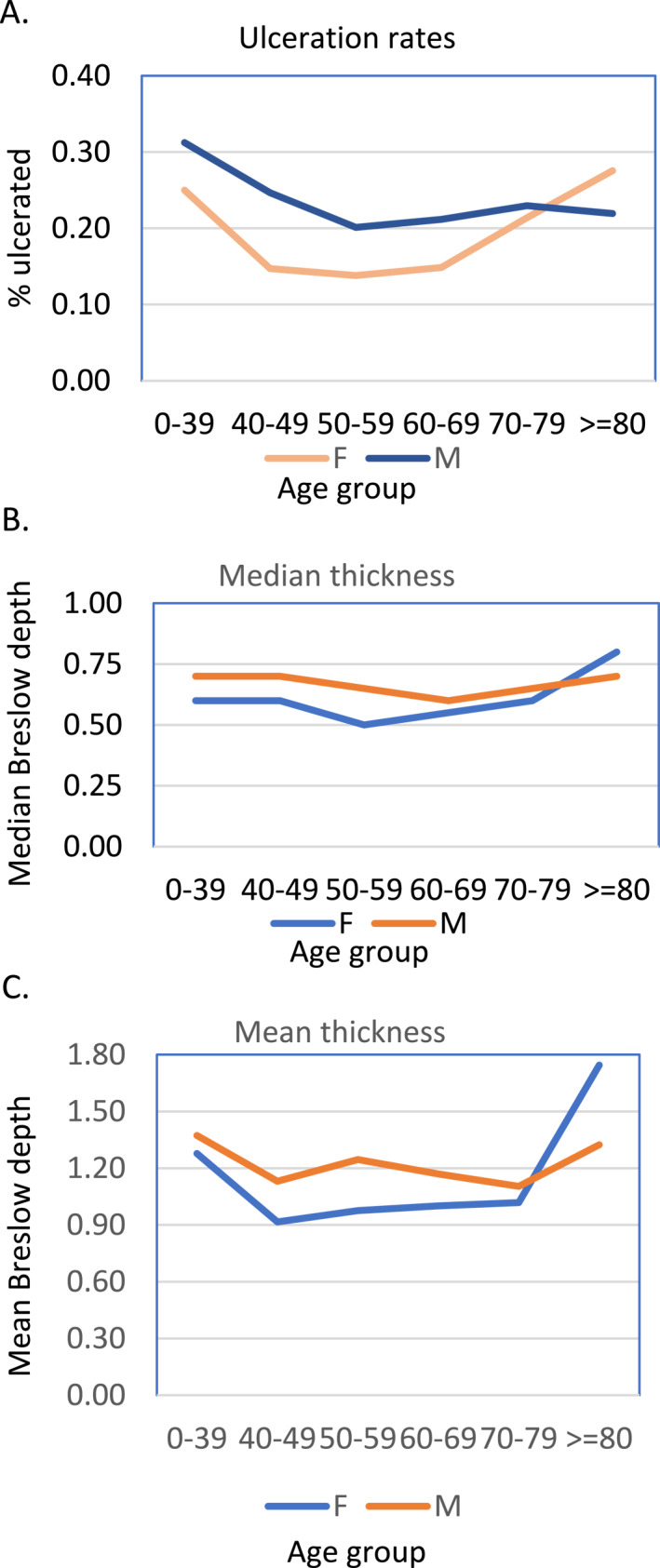
Age‐specific ulceration rate and Breslow depth of invasive melanoma in the University of Tennessee Medical Group dataset. (a) Percentage of ulcerated melanoma in each age group in either sex. (b and c) The median (b) and mean (c) Breslow depth of melanoma in each age group.

There is no sex difference in each age group on the ulceration rate (bottom half of Table 2, *p* values on the right column). Not all age groups have the same ulceration rate in women (*p* = 0.018); this difference is not present in men (*p* = 0.77) (Table 2). For females, ulceration rates follow a ‘U’ shaped curve with a greater rate of ulceration at the younger and older age groups (Figure 1a), which is not apparent in men. Tumours in women in the age group of 40–69 showed the lowest ulceration rate (Figure 1a).

### 3.4 Breslow depth

Of the 2318 invasive melanomas, Breslow depth was reported for 2132 cases. Rank‐sum analysis revealed a significant difference in the Breslow depth between men and women with a *p*‐value <0.001 (Table 3) but no sex difference was observed for each site (Table 3, *p* values on the right column). There is no site difference in the Breslow depth in women, but the difference is significant in men (*p* = 0.009, Kruskal–Wallis rank‐sum test, Table 3). The Breslow depth is the thickest in men's tumours located in the buttock/groin area (median 2.03 mm), compared to all tumours in men (median 0.65 mm). The next thickest tumours are those located on men's foot (median 1.25 mm). A similar observation also occurred in women, with a median Breslow depth of 1.5 mm for tumours on feet, much higher than the median of all tumours in women (0.60 mm).

**TABLE 3:** Site and age differences in Breslow depth.

	Female				Male				Total				*p* value^a^
	*n*	Mean	Median	IQR	*n*	Mean	Median	IQR	*n*	Mean	Median	IQR	
Site													
Scalp	10	1.70	1.15	1.1	110	1.73	1.08	1.45	120	1.73	1.09	1.43	0.92
Face	48	1.14	0.60	1.02	114	1.23	0.8	0.83	162	1.20	0.70	0.90	0.23
Ear	11	1.68	0.70	0.60	74	1.04	0.72	0.80	85	1.12	0.70	0.80	0.08
Neck	23	0.95	0.40	0.68	64	1.00	0.58	1.08	87	0.99	0.52	1.03	0.36
Chest	45	0.73	0.50	0.50	73	1.45	0.51	0.90	118	1.17	0.50	0.62	0.08
Abdomen	21	1.35	0.55	0.50	37	0.86	0.60	0.60	58	1.04	0.58	0.50	0.88
Back	177	1.21	0.52	0.50	373	1.15	0.60	0.63	550	1.17	0.60	0.60	0.12
Upper arm	184	1.19	0.60	0.79	252	1.10	0.60	0.73	436	1.14	0.60	0.77	0.72
Forearm/hand	66	1.09	0.60	0.85	130	1.05	0.60	0.70	196	1.06	0.60	0.80	0.33
Buttock/groin	14	0.94	0.60	0.74	6	2.09	2.03	1.65	20	1.28	0.80	1.42	0.06
Thigh/hip	85	0.75	0.50	0.55	29	0.93	0.50	0.70	114	0.80	0.50	0.50	0.41
Lower leg	100	0.86	0.60	0.62	47	1.31	0.65	1.10	147	1.01	0.60	0.70	0.20
Foot	24	2.15	1.50	2.67	10	1.34	1.25	0.80	34	1.91	1.35	1.60	0.75
Total	808	1.10	0.60	0.70	1319	1.19	0.65	0.85	2127	1.16	0.60	0.80	**<0.001**
*p* values^b^	0.41				**0.009**				**<0.001**				
Age													
0–39	90	1.28	0.60	0.70	41	1.37	0.70	0.93	131	1.31	0.66	0.80	0.31
40–49	108	0.92	0.60	0.54	77	1.13	0.70	0.75	185	1.01	0.60	0.70	**0.024** **^c^**
50–59	153	0.98	0.50	0.58	193	1.25	0.65	0.77	346	1.13	0.60	0.70	**0.0004**
60–69	207	1.00	0.55	0.60	385	1.17	0.60	0.90	592	1.11	0.60	0.79	**0.007**
70–79	151	1.02	0.60	0.72	415	1.10	0.65	0.80	566	1.08	0.62	0.8	0.28
≥80	103	1.75	0.80	2.10	209	1.32	0.70	0.76	312	1.46	0.70	1.24	0.17
Total	812	1.11	0.60	0.70	1320	1.19	0.65	0.85	2132	1.16	0.60	0.80	**0.0003**
*p* values^b^	0.41				0.51				**0.030**				

*Note*: Bold values indicate the significant p values, with two‐sided alpha 〈0.05.

^a^ *p* values indicate sex difference on each site or in each age group, based on the Wilcoxon rank‐sum test.

^b^ *p* values indicate site or age difference, based on the Kruskal–Wallis rank‐sum test.

^c^ *p* value is not significant if the Bonferroni correction is applied (*α* = 0.0083).

There is a significant difference in the Breslow depth between males and females for the age groups of 40–49, 50–59, 60–69 (Table 3, note that the 40–49 group lost significance after multiple comparison corrections), but not for the youngest and oldest groups (<40 and >70). Males have a thicker tumour burden in these 40–69 age groups (Figure 1b,c). This difference was not observed for the youngest age group or the older age groups.

### 3.5 RTD on each site

Using the average percentage distribution of BSA for each site of US men and women from previous studies,^21^, ^23^ the RTD on each of the anatomic site (in situ and metastatic tumours are all included) was calculated by dividing the percentage of case numbers at each body site by an average percentage of BSA of that site.

As shown in Table 4, for men, the top three sites with the highest density are the ear, face and neck. For women, it is the face, ear, and upper arm and all upper body. The three sites with the least density are the buttock/groin, thigh/hip and foot for men, and buttock/groin, foot and scalp for women. The sex difference in RTDs (M/F ratios of RTDs, last column in Table 4) varies greatly on different sites. As compared to women, the scalp, ear, neck and face in men exhibit much higher RTDs, whereas the foot, buttock/groin, lower leg and thigh/hip are much lower. Scalp and thigh/hip are two sites with the greatest sex difference as the ratios reach 7–8 (men to women ratio is 7.1 for scalp, women to men ratio is 7.9 for thigh/hip).

**TABLE 4:** Relative tumour density on each site in each sex.

	Order	Location	Female%	Male%	%BSA	RTD‐F	RTD‐M	M/F
UMTG data	1	Scalp	1%	8%	3.7	0.30	2.11	7.09
	2	Ear	1%	7%	0.5	2.80	14.00	5.00
	3	Face	13%	18%	2.4	5.21	7.63	1.46
	4	Neck	4%	7%	2.4	1.46	3.08	2.11
	5	Upper arm	22%	16%	8.0	2.79	1.98	0.71
	6	Back	19%	23%	10.0	1.94	2.28	1.18
	7	Chest	7%	7%	10.0	0.70	0.68	0.97
	8	Forearm/hand	9%	8%	11.0	0.83	0.75	0.90
	9	Abdomen	2%	2%	6.0	0.38	0.28	0.74
	10	Buttock/groin	0.7%	0.2%	6.0	0.12	0.03	0.29
	11	Thigh/hip	9%	1%	19.0	0.46	0.06	0.13
	12	Lower leg	10%	2%	14.0	0.69	0.16	0.23
	13	Foot	2%	0.6%	7.0	0.29	0.09	0.30
SEER data		Scalp_neck				0.41	0.95	2.32
		Ear				1.14	4.33	3.79
		face				1.98	2.35	1.18
		Arm/shoulder				0.52	0.37	0.72
		Trunk				0.79	1.01	1.28
		Thigh/leg				0.42	0.11	0.27

Abbreviations: BSA, body surface area; M/F, RTD‐M/RTD‐F (ratio); RTD‐F, RTD for females; RTD‐M, RTD for males; SEER, Surveillance, Epidemiology and End Results Program.

The face and ear exhibit the highest density in our dataset, but not the scalp. Women's scalp does not show a particularly high RTD. We used the SEER data for validation on this observation. Melanoma cases from the SEER data (1992–2020) were extracted and percentage of tumours in each site were calculated, which were then divided by each site's BSA in m^2^ as calculated by the above‐mentioned percentage of BSA and total BSA (2.03 m^2^ for men and 1.78 m^2^ for women).^23^ The data are listed in Table 4 for comparison. Indeed, the face and ear have the highest RTDs in both sexes, far exceeding other sites (Table 4). Sites with the least densities are the thigh/leg and arm/shoulder for men and the thigh/leg and scalp for women.

**FIGURE 2:**
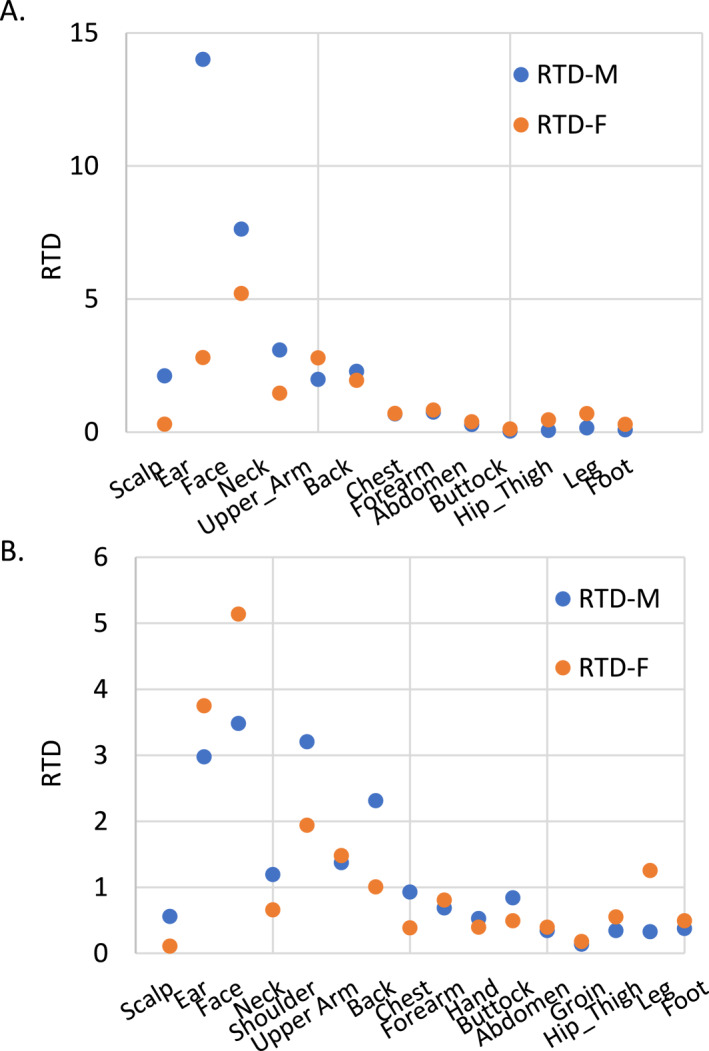
Scatter plot showing the moderate head to toe gradient of melanoma density. (a) The University of Tennessee Medical Group dataset and (b) the Bulliard et al. dataset^21^ (plotted according to data presented in Table 1 of the original paper).

When the anatomic sites are ordered roughly in a head‐to‐toe manner, as shown in Table 4, with scalp as 1 and foot as 13, and the RTDs are plotted using this ranked order as the *X*‐axis, there is a moderate fit into a linear regression model (*R* ^2^ is 0.45 for men and 0.33 for women, Figure 2a). To validate this linear regression, data from Bulliard et al. (2007) were used to generate a similar plot. The linear fitting is also moderate, with *R* ^2^ of 0.44 for men and 0.24 for women (Figure 2b). This head‐to‐toe gradient is also observed in the SEER data, despite the sites are not refined as our data. As shown in Table 4, the highest RTD is observed in the ear and face, while the lowest is in the thigh/legs, with the only exception being the RTD of the female scalp, which is close to that of the female thigh/leg.

## 4 DISCUSSION/CONCLUSION

The present study aimed to provide a detailed analysis of the anatomic distribution of CMM and its correlation with age, sex and tumour characteristics such as the Breslow depth and ulceration status. The data were obtained from a single academic institution and involved a total of 7236 primary and metastatic CMM cases from 2013 to 2021. Our study confirmed significant sex differences regarding tumour characteristics and body location, which is consistent with previous reports. Taking advantage of the fine records of the body location of tumours, we further revealed that the RTD showed a moderate head‐to‐toe gradient pattern, and with the ear and face showing the highest RTDs in men and women, it may require additional attention for prevention.

In terms of tumour characteristics, consistent with the literature, males were more likely to have ulceration than females, which is one of the most powerful predictors of melanoma survival.^24^, ^25^ Men had higher rates of ulceration across all age categories until the age ≥80. Interestingly, younger and older women had higher rates of ulceration than middle‐aged women, thus the ulceration rate forms a ‘U’ shape along the age axis. This is different from most reported results which show a general trend of increasing ulceration and Breslow depth of tumours with age.^26^ However, it is consistent with our dataset that also showed thinner tumours in the 50–79 age groups. Similar to ulceration, the Breslow thickness also carries a significantly increased mortality risk, especially among older adults.^27^

When taking the BSA into consideration, the tumour distribution seems to exhibit a rough head‐to‐toe gradient, with some exceptions. The ear and face are the two sites showing the highest RTDs than other sites in both sexes, but the scalp is inconsistent in the two sexes, with a relatively high RTD in men but a relatively low RTD in women. This discrepancy may be caused by the protective effect of longer hair in women.^28^

In the trunk, RTDs in the back are higher than those in the front including the chest and abdomen, in both men and women. RTDs in the upper arm are higher than those in the forearm and hand in both men and women. The buttock/groin and foot are the two sites among the lowest RTDs in both sexes (men's thigh/hip is another site), even though one may speculate that the buttock/groin is rarely exposed to UV while the foot is more exposed. The thigh/hip and lower leg also exhibit very low RTDs in men but a relatively middle level in women. These results are consistent with our recent discovery which showed that men's legs show a remarkably lower susceptibility to melanoma, while women's melanoma distribution is relatively more even among anatomic sites.^17^ According to limited literature, the legs seem to be more exposed to the sun as compared to arms.^29^ In additional, occupational risk may also give differential non‐UV exposure to legs and arms. For example, chimney sweepers are more likely to develop CMM in their upper arms than the general population.^30^ These reasons may contribute to the observed difference in legs and arms.

A limitation of this study is that the average BSA of US men and women was used in density calculation, and hence, the accuracy was compromised. It is possible to calculate individual BSA using height and weight or the modern three‐dimensional scanning technology,^31^ which could provide better precision of density calculation.

Additionally, information regarding outdoor activities and potential carcinogen exposure was not available, hence the impact of these risk factors is not counted for in this study. It was reported that men with outdoor work had a low standardized incidence ratio of 0.79,^32^ and exposure to crude oil or benzene can increase risks of CMM on the forearm and hand among those of the offshore workers.^33^ Furthermore, melanoma is also a possible occupational disease among firefighters with a history of exposure^34^ yet this information was also not available in our dataset.

Solar UV radiation received by individuals is highly variable by the season, outdoor activity types, clothing and sunscreen use and anatomic sites. There are a number of studies showing the dosimeter‐measured UV doses received at various anatomical sites. In general, upper body sites such as the head, shoulder, arms, chest and back receive more UV radiation than the lower body, such as the thigh and legs.^35^, ^36^ Vertex is normally the site that receives the highest UVR as it is horizontal, but the face only receives about half of the amount for the vertex.^37^ Therefore, hair perhaps provides significant protection against UVR for the scalp and ear in women,^28^ especially the scalp, which did not show a particularly high RTD. Instead, uncovered ears in men showed the highest RTD. The face also shows high RTDs in both sexes as this part is normally exposed.

Overall, the discrepancy in RTDs in different body sites may largely be explained by UV exposure, but there are apparent exceptions. For example, the RTDs in the legs are much lower than those from the arms (including the upper arm and forearm), despite that the legs perhaps have an equal opportunity to be exposed to UV as the arms. In a standing position when a person is facing the sun, the upper body apparently receives more UVR than the lower body in general, but the arms seem to receive similar levels of UVR as the legs.^38^

In addition to UVR, it is largely unknown whether or how the body distribution of physiological factors affects melanoma development. It is known that the melanocyte density in anatomic sites varies,^21^ which is a potential impacting factor as melanomas arise from melanocytes or their precursor cells. The subcutaneous fat may have an impact on melanoma development as well, as previous reports showed that the ratio of visceral fat over the subcutaneous fat is associated with melanoma patient survival and fat cells can stimulate metastasis.^39^, ^40^ We have recently shown that the tumour androgen receptor level is positively associated with melanoma patient survival,^41^ suggesting a role of sex hormones in melanoma development. It is suggestive in the literature that sex hormones and their receptors are not uniformly present on different anatomic sites, as previous studies found an androgen receptor expression on the genital skin but not in other areas.^42^ The roles of host biological factors in melanoma development guarantee further investigation to ensure a better understanding of melanoma development, and hence, a better prevention base.

## CONFLICT OF INTEREST STATEMENT

The authors have declared no conflicts of interest.

## AUTHOR CONTRIBUTIONS

**Meryl Musicante**: Formal analysis (equal); investigation (equal); writing—original draft (equal). **James Ferrer**: Data curation (equal); investigation (equal); writing—original draft (equal); writing—review & editing (equal). **Jianjian Lin**: Data curation (equal); formal analysis (equal). **Tejesh Surendr Patel**: Conceptualization (equal); data curation (equal); supervision (equal); writing—review & editing (equal). **Feng Liu‐Smith**: Conceptualization (equal); data curation (equal); formal analysis (equal); investigation (equal); methodology (equal); supervision (equal); validation (equal); visualization (equal); writing—review & editing (equal).

## ETHICS STATEMENT

The UTHSC IRB approved our study to extract de‐identified information of patients from our medical records.

## PATIENT CONSENT

Not applicable.

## Supporting information

Table S1

## Data Availability

The data underlying this article will be shared with the corresponding author on reasonable request.
