# Supplementary material for: A retrospective single‐institute study reveals a vertical gradient of the density of cutaneous melanoma from head to toe
Source: Skin Health Dis. 2024 Nov 2;4(6):e463. doi: 10.1002/ski2.463 (PMC11608879; doi:10.1002/ski2.463)
Supplement: Supplementary file 1 — Table S1 [file SKI2-4-e463-s001.docx]

| Supplemental Table S1: age-specific distribution of melanoma in men and women | | | | | | | |
| --- | --- | --- | --- | --- | --- | --- | --- |
|  | Females | | Males | | Total |  |  |
| Age Group | n | % | n | % | n |  |  |
| 0-39 | 233 | 76 | 74 | 24 | 307 |  |  |
| 40-49 | 314 | 62 | 196 | 38 | 510 |  |  |
| 50-59 | 530 | 48 | 563 | 52 | 1,093 |  |  |
| 60-69 | 768 | 37 | 1,328 | 63 | 2,096 |  |  |
| 70-79 | 592 | 28 | 1,502 | 72 | 2,094 |  |  |
| >=80 | 320 | 28 | 816 | 72 | 1,136 |  |  |
| Total | 2,757 | 38 | 4,479 | 62 | 7,236 |  |  |
